# Advertising of Human Milk Substitutes in United Kingdom Healthcare Professional Publications: An Observational Study

**DOI:** 10.1177/08903344211018161

**Published:** 2021-05-16

**Authors:** Natalie Hickman, Sarah Morgan, Helen Crawley, Marko Kerac

**Affiliations:** 1 4906 Department of Population Health, London School of Hygiene & Tropical Medicine, London, UK; 2 First Steps Nutrition Trust, London, UK; 3 London, Kent, Surrey and Sussex School of Public Health, London, UK; 4 Centre for Maternal, Adolescent, Reproductive and Child Health, London School of Hygiene & Tropical Medicine, London, UK

**Keywords:** bottle feeding, breastfeeding, breastfeeding barriers, breastfeeding knowledge, human milk substitute, infant formula, International Code of Marketing of Breast-Milk Substitutes, media analysis, public health

## Abstract

**Background:**

Inappropriate marketing of human milk substitutes negatively influences efforts to protect breastfeeding. Although healthcare professionals can positively influence infant feeding decisions, government regulations permit manufacturers to communicate messages to them through advertising.

**Research Aims:**

(1) To identify the extent of human milk substitute advertising in publications aimed at United Kingdom healthcare professionals and (2) to describe compliance with the International Code of Marketing of Breast-Milk Substitutes and United Kingdom Formula and Follow-on Formula Regulations.

**Methods:**

This was a cross-sectional observational study. We reviewed publications targeting healthcare professionals working with families in the United Kingdom (*N* = 19). Quantity and type of human milk substitute advertisements, as a proportion of all advertising, in each publication were recorded. All unique advertisements were double assessed for compliance.

**Results:**

Human milk substitute advertising was found in nine (47%) of the 19 publications (four affiliated with professional associations), making up 10.3% (*n* = 196) of these publications’ total advertising. Of human milk substitute advertisements found, 65.4% (*n* = 110) were for products used to manage cows’ milk protein allergy. Of the 32 unique human milk substitute advertisements found, none complied with the International Code of Marketing of Breast Milk Substitutes or United Kingdom Formula and Follow-on Formula Regulations.

**Conclusions:**

Many healthcare professionals’ publications had non-compliant human milk substitute advertisements. There is an urgent need to ensure full compliance with international and local regulation in future advertisements and to consider whether advertisements are justified at all.

## Background

The United Kingdom (UK) has some of the lowest breastfeeding rates in the world, the reasons for which are complex and multifactorial. Evidence has shown that one important global barrier to breastfeeding is inappropriate marketing of human milk substitutes (HMS; McFadden et al., 2016). The marketing of HMS to healthcare professionals (HCP) who support families with infants contributes to a lack of awareness and understanding about the physiological process of breastfeeding, increases social barriers by normalizing HMS use, and makes breastfeeding an unattainable goal for many women (Grummer-Strawn & Stahlhofer, 2018; Rollins et al., 2016). While many midwives and health visitors in the UK receive training through the United Nations Children’s Fund (UNICEF) UK Baby Friendly Hospital Initiative, some HCP receive limited pre-registration training about breastfeeding (Biggs et al., 2020; World Breastfeeding Trends Initiative, 2016).

The International Code of Marketing of Breast-Milk Substitutes (IC; World Health Organization [WHO], 1981) and the subsequent relevant World Health Assembly resolutions (WHO, 2018) provide recommendations for the marketing of HMS. The WHO (1981) has defined “breast-milk substitute” [HMS] as “any food being marketed or otherwise presented as a partial or total replacement for breast milk, whether or not suitable for that purpose” (p. 13). This definition therefore encompasses infant formula, follow-on formula, specialist formula (regulated as foods for special medical purposes [FSMP] in the European Union [EU]) and milks marketed for children over 1 year of age (WHO, 2016). Wording in the IC clarifies that HCP are an important source of information and support for infant feeding practices and that they should be able to provide, if necessary, unbiased information on the proper use and safe preparation of infant formula (WHO, 1981). The IC specifies that “information provided by manufacturers and distributors to health professionals regarding products within the scope of this Code should be restricted to “scientific and factual matters” (WHO, 1981, p. 12) and sets out requirements for countries to use in creating legislation. While almost all countries have signed up to the IC, few have fully adopted all the recommendations into law (WHO, 2020). EU regulations incorporate some provisions of the IC into law for all member states, which implement this in their domestic legislation. When the data were collected for this study, the UK legislation relating to composition, marketing and labelling of infant formula, follow-on formula and FSMP were Statutory Instruments acted in all four countries of the UK mirroring *EU Directives 2006/141/EC* and *1999/21/EC* and any subsequent amendments (The Infant Formula and Follow-on Formula [Wales] Regulations, 2007; The Infant Formula and Follow-on Formula [England] Regulations, 2007; The Infant Formula and Follow-on Formula [Scotland] Regulations, 2007; The Infant Formula and Follow-on Formula [Northern Ireland] Regulations, 2007; The Medical Food [England] Regulations, 2000; The Medical Food [Wales] Regulations, 2000; The Medical Food [Northern Ireland] Regulations, 2000; Foods for Special Medical Purposes [Scotland] Regulations, 2000). The infant formula and follow-on formula legislation permits advertising of infant formula in scientific and trade publications (where the audience is not the general public) and specifies that the advertisements only contain information of a “scientific and factual nature” UK of Health, 2013, p. 29). While this mirrors the wording in the IC, there is no mechanism in UK law to challenge or review any scientific and factual information provided by HMS manufacturers, making this wording inconsequential. Regulations for FSMP in the UK at the time we conducted this research had no restrictions related to product marketing. There is, however, specific wording in both the IC and UK legislation that can be used to assess advertisements’ compliance with the spirit of the IC and UK regulations, and this has been summarized by Morgan et al. (2018).

**Key Messages**

- There is limited evidence on the quantity and content of advertisements for human milk substitutes in publications aimed at United Kingdom healthcare professionals.
- Human milk substitute advertisements were found in nine of the 19 (47%) publications searched, including four affiliated with professional associations.
- Of the 32 unique human milk substitute advertisements found, none complied with the International Code of Marketing of Breast-Milk Substitutes or UK Formula and Follow-on Formula Regulations 2007.
- Widespread, but non-compliant, human milk substitute advertisements in healthcare professional publications risk undermining efforts to protect and promote breastfeeding.

Previously, advertising of HMS to HCP in the UK has been reviewed and the “scientific and factual” data presented in the advertisements challenged as being misleading or not in line with findings from independent policy makers, as well as containing emotive wording and imagery (Crawley & Westland, 2016; Westland & Crawley, 2019). The quantity and content of HMS advertisements in UK medical journals has also been reviewed. Although, only a small proportion of HMS advertising (1.7%) was found and IC compliance among reviewed publications containing HMS advertising was assessed as poor (Morgan et al., 2018). Concerns about the influence of HMS advertising have been highlighted by others (Costello et al., 2017; Morgan et al., 2019; Waterston & Wright, 2019) and this has contributed to some professional associations and publications reviewing and amending their policies on accepting advertising and funding from HMS companies (Godlee, 2019; Royal College of Paediatrics and Child Health, 2019). While some HCP may argue that they can easily distinguish between the sales pitch and the facts, no one is immune to the persuasive power of marketing (Hastings et al., 2020). Through this study we aimed (1) to identify the extent of human milk substitute advertising in publications aimed at UK HCP and (2) to describe compliance with the IC and UK Regulations.

## Methods

### Design

This was a cross-sectional observational study. We chose this as the most appropriate design to determine the quantity of HMS advertisements found in HCP publications over a period of 1 year. The study protocol was approved by the ethics board at the London School of Hygiene and Tropical Medicine.

### Setting and Relevant Context

Healthcare in the UK is free at point of use and provided universally, based on clinical need, by the National Health Service (NHS). The NHS is publicly funded through taxation in the UK and incorporates the four individual healthcare services of England, Wales, Scotland, and Northern Ireland. Pregnant women and new mothers in the UK are predominantly supported by midwives and health visitors, although additional support is provided by general practitioners (GPs), pediatricians, and nurses over the course of pregnancy and in the first 5 years of the child’s life. The majority of midwifery and health visiting services in the UK are either UNICEF Baby Friendly Hospital Initiative accredited or working towards accreditation. However, families may seek or be offered information and support from GPs, nurses, pediatricians, or dietitians, especially if there are complications or problems with infant feeding. The UK no longer routinely collects data on infant feeding, after the last *UK Infant Feeding Survey* conducted in 2010. Breastfeeding data are currently collected by each country individually at different timepoints and using different methods. NHS England reports breastfeeding initiation rates of 57% (NHS Digital, 2021), with 48% of babies reported as receiving any human milk at their 6–8-week appointments with a health visitor (Public Health England, 2021). Public Health Northern Ireland reports that 49% of infants are receiving human milk on discharge from maternity services, reducing to 33% at 6 weeks (Public Health Intelligence Unit, 2019). Exclusive breastfeeding rates are recorded in Wales as 55% at birth, reducing to 25% at 6 weeks (Betsi Cadwaladr University Health Board, 2019). Scotland is the only UK country where an infant feeding survey is conducted, and breastfeeding rates were 55% at 6 weeks (Healthier Scotland, 2017).

### Sample

Journals and magazines available to UK-based midwives, health visitors, dietitians, pediatricians, GPs, and nurses through professional associations, or privately published, between August 2018 and July 2019 were reviewed. We chose these HCP groups because they have direct contact with pregnant women, infants, and young children and are therefore in a position to advise and potentially influence behavior through that advice. Publications targeting the HCP groups specified were selected for inclusion if they were automatically delivered as part of membership to an association, free magazines, and other well-known magazines or journals available for the specified HCP groups. At least two publications for each HCP area were selected, with at least one affiliated with a professional association. Publications containing no advertising material were excluded from the study.

For inclusion in the study, selected journals or magazines needed to be available as either hard copies or as full digital copies. To ensure consistency with the number of issues searched and the total number of issues published over a year, unavailable issues were replaced with the next available issue prior to the start of the data collection period. In total, 19 publications targeted for the six different HCP groups were included in the study, 10 of these were affiliated with professional associations. The number of issues of each publication varied, with the least number of issues from journals published quarterly (*n* = 4) and the most from those published weekly (*n* = 46). In total, 207 issues of all publications were searched. As no statistical analysis of the data collected for this study was planned, we did not conduct a sample size calculation.

### Measurement

#### Aim 1: Quantity and Type of HMS Advertising

To determine the quantity of advertising, an existing data collection tool created by Morgan et al. (2018) was adapted for the collection of the following information for each issue of the journals or magazines included in the study: number of pages (defined as one side of a physical page in the publication), number of pages given to advertisements, number and type of different advertisements, number and type of different HMS advertisements for infants under 6 months, number of different advertisements for follow-on or toddler milk, number of pages given to HMS advertising for infants under 6 months, and number of pages given to advertising for follow-on or toddler milk. All printed content was included in the page count including front and back covers. All promotional material was counted as advertising, including sponsored articles, information about courses, advertisements for events with clear sponsorship information, articles detailing award recipients and events with clear sponsorship information, advertisements for the publication or the professional association with which the publication was affiliated, and calls for submissions. Fractions of pages of advertising were rounded to the nearest sixth of a page. Advertisements covering more than one page were counted as the same advertisement if for the same product and each page (one side of a physical page) was counted in the page count. Different advertisements for the same product were counted as separate advertisements if there was at least one clear page between them. Clearly designated recruitment or classified sections were not counted as advertising but were included in the page count. Articles sponsored by industry were counted as advertisements. For industry-sponsored articles covering several pages, all pages were counted in the page count of advertisements whether the sponsorship was clear on all pages or not.

Advertisements for all types of HMS were counted as HMS advertisements including infant formula, follow-on formula, toddler milks/growing up milks, specialist milks designated as FSMP for infants under 6 months, specialist milks designated as FSMP for infants 6 months and over, and advertorials for a specific brand of HMS. Sponsored articles by HMS companies covering a topic but not advertising a specific product were not counted as HMS advertising but were counted as sponsored articles in the advertising count. HMS were categorized using information on brands and infant milk types marketed in the UK collated by First Steps Nutrition Trust (www.firststepsnutrition.org). Each issue of each publication was primarily reviewed, and data recorded in the data collection tool using Microsoft Excel (2019). To ensure validity, each issue was then re-reviewed and recorded data were confirmed as accurate. In the event of any discrepancies during the second review, a third review of the issue was undertaken.

#### Aim 2: Compliance of HMS Advertisements

All unique HMS advertisements found were assessed by two of the authors independently using the data collection tool created by Morgan et al. (2018). To determine compliance with the IC, HMS advertisements were assessed against measures relating to items specified in Article 7.2 (WHO, 1981; Table 1). Additional objective measures were added to the data collection tool based on points from the Department of Health Guidance Notes on the Infant Formula and Follow-on Formula Regulations, 2007, covering information on advertising to HCP (United Kingdom Department of Health, 2013). These measures were used to assess HMS advertisements for compliance with UK regulations. Any discrepancies in the outcomes of specific advertisement assessments were discussed by the authors who conducted the assessments in order to reach an agreement.

**Table 1:** Variable Definitions and Measurement.

Variable	Definition	Reference	Measured by
Advertisements with only promotional statements	Assess advertisement as ‘scientific and factual’	WHO, 1981, Article 7.2, p. 12., United Kingdom Department of Health, 2013, Appendix IV, p. 29	Total *n* of statements *n* of promotional statements *n* of statements with supporting references *n* of references
Advertisements with statement(s) expressing equivalence/similarity to human milk.	Assess compliance with IC Article 7.2	WHO, 1981, Article 7.2, p. 12	Contains statement(s) with wording expressing similarity /equivalence/likeness to human milk or breastfeeding (yes/no) List statement(s) with wording expressing similarity/equivalence/likeness to human milk or breastfeeding Contains statement that HMS is superior to human milk or breastfeeding? (yes/no) List statement(s) with wording expressing similarity/equivalence/likeness to human milk or breastfeeding
Different HMS advertisements with statement of superiority of human milk	To assess compliance with provision of ‘Important Notice’	United Kingdom Department of Health, 2013 Paragraph 27, p11 & Paragraph 57, p17	Is there a statement on the benefits and superiority of breastfeeding? (yes/no) Is 'Important Notice' included (concerning the superiority of breast feeding and advice on when infant formula should be used) (yes/no)
			If included, is the important notice afforded a high degree of prominence/ clearly visible? (yes/no)
Advertisements compliant with United Kingdom Infant Formula and Follow-on Formula Regulations 2007.	Assess compliance with all criteria specified in UK Regulations	United Kingdom Department of Health, 2013, Paragraph 47 & 48, p. 15-16, Paragraph 27, p. 11	Compliant with UK Infant Formula and Follow-on Formula regulations 2007? (yes/no) ‘Important Notice’ must be present Must answer ‘no’ to the following questions: If the product advertised is follow-on or growing up milk, is it unclear from the text and images that the product advertised is for older babies? (yes/no) Does the advertisement promote a range of products by making the brand the focus of the advertisement, rather than specific products? (yes/no) Does the advertisement feature text or images which relate to pregnancy or the feeding or care of infants under 6 months? (yes/no) Does the advertisement include pictures or text that directly or indirectly relate or compare products to human milk? (yes/no) Does the advertisement focus on carers emotions in relation to the feeding or care of infants under six months? (yes/no) Does the advertisement feature babies that consumers may perceive as being under 6 months (even if they are over 6 months)? (yes/no) Does the advertisement focus primarily on the promotion of ingredients, or the effect of ingredients, which are common to both follow-on formula and infant formula? (yes/no)
Advertisements compliant with the IC	To assess compliance with all criteria specified in IC	WHO, 1981, Article 4.2, p10 & Article 7.2, p. 12	Advertisement is compliant with IC (yes/no) Contains statement(s) with wording expressing similarity/equivalence/likeness to human milk or breastfeeding (Yes/no) If yes to above, advertisement is not IC compliant Must answer ‘yes’ to the following: Is there a statement on the benefits and superiority of breastfeeding? (yes/no) Is there a statement on maternal nutrition and the preparation for and maintenance of breastfeeding? (yes/no) Is there a statement on the negative effect on breastfeeding of introducing partial bottle-feeding? (yes/no) Is there a statement on the difficulty of reversing the decision not to breastfeed? (yes/no) Does material provide information about the use of infant BMS? (yes/no) If yes answer remaining questions. If no score through boxes. Is there a statement on the proper use of infant BMS? (yes/no) Is there a statement on the social and financial implications of the use of HMS? (yes/no) Is there a statement on the health hazards of unnecessary or improper use of HMS? (yes/no)

*Note.* IC = International Code of Marketing Breast-Milk Substitutes; HMS = human milk substitutes; UK = United Kingdom.

### Data Collection

The selected publications covering the period of August 2018–July 2019 were reviewed. Data about the quantity and type of HMS advertisements were collected by the lead author in July 2019 and, for hard copy publications, this took place at the Royal College of Midwives Library, the British Medical Association Library, and the British Library. For the two publications (*Dietetics Today* and *Network Health Digest*) available as full pdf digital copies, data were also collected in July 2019 by the lead author. Each issue of each publication covering the study period was hand searched and data were recorded in Microsoft Excel (2019). Photographs were taken of all unique HMS advertisements found during data collection and the first assessment of these was undertaken in July 2019 by the lead author, once data from all publications had been collected. The second assessment of each unique HMS advertisement found was conducted by a different author independently, during September and October 2020, using the photographs taken when data were originally collected and also recorded using the same data collection tool in Microsoft Excel (2019).

### Data Analysis

The quantity and extent of HMS advertising (Aim 1) were analyzed using the number of pages of HMS advertising in comparison with the number of pages of all advertising to present the proportion of HMS advertising in each publication and across all publications. For compliance of HMS advertisements (Aim 2), each assessed HMS advertisement was categorized into the variables described in Table 1. The number of HMS advertisements meeting the criteria for each variable was compared with the total number of HMS advertisements to calculate the proportion of HMS advertisements meeting the criteria for compliance with the IC and UK Regulations.

## Results

### Aim 1: Frequency and Type of HMS Advertisements

**Figure 1:**
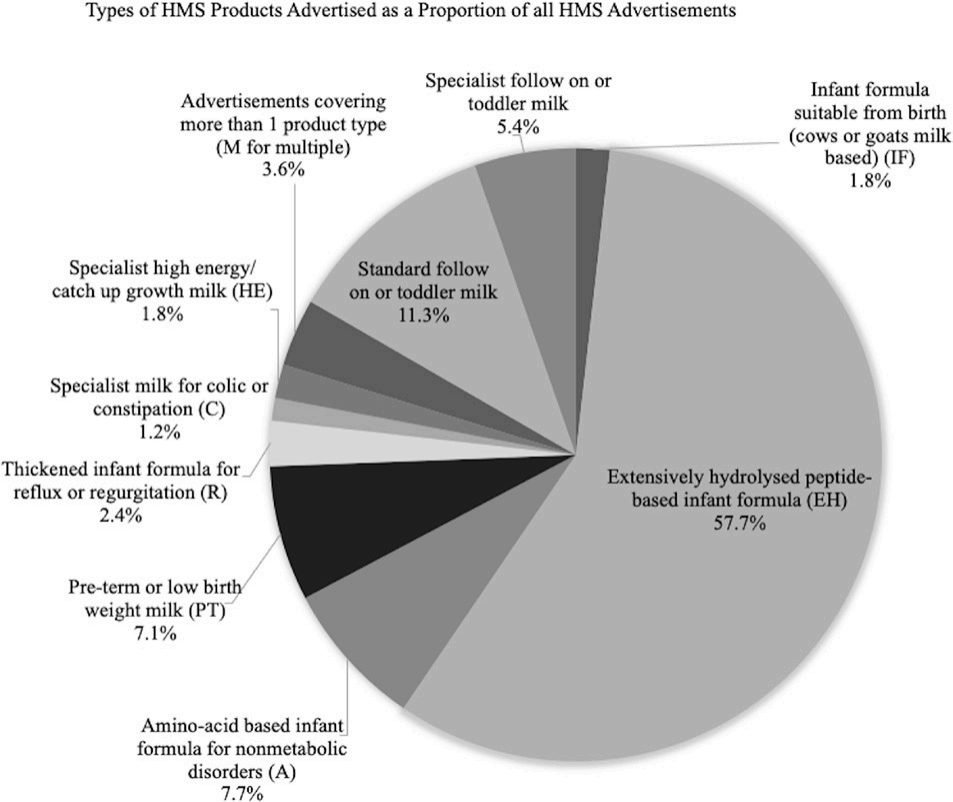
Type of HMS Products Advertised as a Proportion of all HMS Advertisements.

A total of 14,250 pages were searched and, across all publications, all advertising made up 18.4% (*n* = 2618.8 pages). However, among publications containing HMS advertising, the amount of all advertising was higher (20.9%, *n* = 1894 pages). HMS advertisements were found in nine of the 19 (47%) publications searched, making up 7.5% (*n* = 196) of all pages of advertising among all publications searched and 10.3% (*n* = 196) among the nine containing HMS advertisements. Of the 10 professional association affiliated publications, four (40%) contained HMS advertising. Dietitians, GPs, and pediatricians had professional membership association affiliated magazines containing HMS advertising. Table 2 displays the amount of HMS advertising by publication. All issues (*n* = 26, 100%) of each of the three publications aimed at dietitians contained HMS advertisements, with two other publications (*Journal of Health Visiting* and *Pulse*) also containing HMS advertisements in the majority of issues (83%, *n* = 20). Figure 1 illustrates the proportion of HMS advertisements for each different HMS product type among all HMS advertisements found.

**Table 2:** Frequency of HMS Advertising in Selected Publications Grouped by Provider Type.

Type of Provider	Publication	Pages *n* (%)
Pediatricians	*Archives of Disease in Childhood^a^*	7 (12)
	*Archives of Disease in Childhood Fetal & Neonatal Edition^a^*	3 (25)
General Practitioners	*British Journal of General Practice^b^*	0 (0)
	*British Medical Journal^c^*	32 (4.3)
	*Pulse*	15 (5.5)
Dieticians	*Complete Nutrition*	42 (18.6)
	*Dietetics Today^d^*	17 (12.9)
	*Network Health Digest*	42 (33.6)
Health Visitors	*Community Practitioner^e^*	0 (0)
	*Journal of Health Visiting*	26 (12.4)
Nurses	*General Practice Nursing*	0 (0)
	*Nursing Children & Young People ^f^*	0 (0)
	*Nursing Standard ^f^*	0 (0)
	*Nursing Times*	0 (0)
	*Primary Health Care ^f^*	0 (0)
Midwives	*British Journal of Midwifery*	12 (9.8)
	*Midwifery Digest*	0 (0)
	*Midwives^g^*	0 (0)
	*Practising Midwifery*	0 (0)

*Note.* Page is defined as one side of a physical page.

^a^Publications affiliated Royal College of Paediatrics and Child Health.

^b^Publication affiliated with Royal College of General Practitioners.

^c^Publication affiliated with British Medical Association.

^d^Publication affiliated with British Dietetic Association.

^e^Publication affiliated with Unite-Community Practitioner’s & Health Visitor’s Association.

^f^Publications affiliated with Royal College of Nursing.

^g^Publication affiliated with Royal College of Midwives.

### Aim 2: Compliance of Advertisements

None of the HMS advertisements were found to be compliant using the IC tool. No HMS advertisements were found to be compliant with UK Regulations using the adapted tool. Table 3 provides the results of the assessment of HMS advertisements for compliance with the different variables specified to assess compliance with the IC and UK Regulations.

**Table 3:** Assessment of Compliance Human Milk Substitutes Advertisements (*N* = 32).

Outcome Measures	HMS Adverts *n* (%)
Containing only promotional statements	6 (19)
Containing statement(s) expressing equivalence/similarity to human milk	13 (41)
Containing statement of superiority of human milk	20 (63)
Containing statement of superiority of human milk in ‘high degree of prominence’	6 (19)

*Note.* Compliance refers to compliance with the International Code of Marketing Breast-Milk Substitutes (WHO, 1981) and the United Kingdom Department of Health (2013). HMS = human milk substitutes.

## Discussion

While we know that inappropriate marketing by HMS companies can result in devastating consequences for infant health in developing and emerging economies (Barennes et al., 2016; Kean et al., 2017; Save the Children UK, 2018), the health and wellbeing of UK infants also is undermined as a result of HMS advertising. HCP have the opportunity to provide information and support to enable the initiation and continuation of breastfeeding. However, the combination of insufficient breastfeeding training and prevalence of HMS advertising in publications may influence the limited understanding of the physiology of breastfeeding for some HCP, leading to the provision of poor-quality information and support for breastfeeding (Biggs et al., 2020; Rothstein et al., 2019). A sense of loyalty and inherent bias resulting from the acceptance of funding or advertising from HMS manufacturers may further impact HCP ability to ensure the information they impart is evidence-based (Costello et al., 2017; Grummer-Strawn et al., 2019; van Tulleken, 2018; Waterston & Wright, 2019).

The high number of HMS advertisements for products marketed to manage cow’s milk allergies (CMA) illustrate issues raised in an investigation by van Tulleken (2018) into the provision of information on CMA from HMS companies. Marketing of products along with promotion of tools to identify CMA enabled a market for new products and strengthened relationships with HCP (van Tulleken, 2018). Similar to our findings, Morgan et al. (2018) also observed that HMS companies have heavily promoted these products to HCP and there was a concurrent increase in the diagnosis of CMA with prescription of these products increasing by nearly 500% between 2006 and 2016 (van Tulleken, 2018).

Article 11.3 of the IC stipulates that manufacturers should consider themselves responsible for ensuring that their marketing practices adhere to those laid out in the IC, independent of other measures implemented in legislation (WHO, 1981, p. 14). Yet, it appears from our findings, and those of others, that manufacturers are not fulfilling this obligation (Crawley & Westland, 2016; Morgan et al., 2018; Westland & Crawley, 2019). We do not know from our current work whether publications review the content of advertisements once space is purchased. Editors of publications which take advertising for revenue are unlikely to be able to influence the content of advertisements accepted; however, editorial boards can reflect on whether they want to support a journal that accepts HMS advertising and may be able to influence advertising policy. Even so, there are no mechanisms in the UK to establish the accuracy of any information given in HMS advertisements. Monitoring of advertisements to HCP in the UK is done by non-governmental organizations and when there has been breach of the guidance notes, which support regulation relating to this area (for example, if an advertisement does not provide a reference in a peer reviewed publication to support a statement), no action has been taken. Without robust procedures for monitoring and enforcement, UK legislation places HMS manufacturers in a trusted position to regulate their own advertising content.

However, change is occurring. Three of the four publications affiliated with professional associations containing HMS advertising were published by The British Medical Journal Group, which announced in February 2019 that it would no longer be accepting advertisements from HMS companies (Godlee, 2019). The Royal College of Paediatrics and Child Health also made a statement in 2019 that it would no longer accept funding from HMS companies (Royal College of Paediatrics and Child Health, 2019). These announcements were made in the middle of our study period, explaining why we found HMS advertisements in *The British Medical Journal*, *Archives of Disease in Childhood*, and *Archives of Disease in Childhood Fetal & Neonatal Edition*. The British Dietetic Association is now the only professional association reported in this study whose publication continues to accept advertisements from HMS companies in 2021. Given that most dietitians receive limited pre-registration training about breastfeeding (World Breastfeeding Trends Initiative, 2016), it appears that they are at particular risk of exposure to advertising for products, which may undermine their support for breastfeeding.

New regulations specified by EU Directive 609/2013 came into force in February 2020 through new delegated acts, with some changes to regulations relating to the marketing of infant formula, follow-on formula and foods intended for infants and young children, food for special medical purposes, and total diet replacement for weight control (FSMP; Regulation 609/2013, 2013). There are now stronger regulations related to how FSMP can be marketed, bringing them into line with infant formula. However, there is no difference in regulations for how products can be marketed to HCP; it is possible that these additional restrictions on marketing to the general public may prompt HMS companies to focus on advertising to HCP, using them to communicate messages to parents.

To the best of our knowledge, only one previous study, published in a non-peer review journal, has investigated the quantity and compliance of HMS advertisements in publications aimed at UK HCP and only medical journals were investigated (Morgan et al., 2018). We searched a wider range of publications available to a larger group of HCP with the potential to influence infant feeding decisions. Additionally, we searched all issues for each publication, rather than a sample of each, enabling us to ensure that our sample was representative and reducing the impact of issue-to-issue variations.

In order to add evidence to support the strengthening of regulation of HMS advertising, additional studies are needed to evaluate the content of HMS advertisements targeting HCP in order to determine compliance with the new February 2020 UK regulations. Future researchers also should focus on the influence of HMS advertising on HCP knowledge and how this translates into practice. Additionally, given the increasing prevalence of online publications and resources, research is needed to investigate the prevalence of digital HMS advertising, which would help provide a more comprehensive picture of the way in which HCP are targeted by HMS companies.

### Limitations

Data for this study were collected in 2019, reflecting the content of publications from 2018–2019 and may not be reflective of the current quantity and content of HMS advertisements. Although we have targeted key publications relevant to HCP working directly with families with infants, these may under-represent what HCP actually read, and other publications, which also may influence them, could have been omitted. We also focused on print media, which each HCP is likely to receive in hard copy but did not review e-versions of each publication or websites for either the publications or the affiliated professional associations—advertising in these venues is likely to differ. We also cannot know whether advertisements identified directly influenced HCP subsequent infant feeding discussions or decisions.

## Conclusion

If publications do carry HMS advertising, at the very least they should comply with the IC and UK Regulations. The actions of *The British Medical Journal* and The Royal College of Paediatrics and Child Health highlight the possibility for professional associations to review and change policy, ensuring that they remain true to their core values to promote health, despite potential financial losses. As this study shows through the 10 publications that choose not to advertise any HMS, yet seem to be surviving and thriving, formula manufacturers’ money is not critical to publication success. What is critical is that infants have the best possible start in life. This involves optimal decisions about feeding. Advertisements are not and can never be an appropriate source of clinical information.
